# The Impact of Temperature on the Risk of COVID-19: A Multinational Study

**DOI:** 10.3390/ijerph18084052

**Published:** 2021-04-12

**Authors:** Hsiao-Yu Yang, Jason Kai Wei Lee

**Affiliations:** 1 Institute of Environmental and Occupational Health Sciences, College of Public Health, National Taiwan University, Taipei 10055, Taiwan; 2 Department of Public Health, College of Public Health, National Taiwan University, Taipei 10055, Taiwan; 3 Department of Environmental and Occupational Medicine, National Taiwan University Hospital, Taipei 100225, Taiwan; 4 Human Potential Translational Research Programme, Yong Loo Lin School of Medicine, National University of Singapore, Singapore S117597, Singapore; phsjlkw@nus.edu.sg; 5 Department of Physiology, Yong Loo Lin School of Medicine, National University of Singapore, Singapore S117593, Singapore; 6 Global Asia Institute, National University of Singapore, Singapore S119076, Singapore; 7 N.1 Institute for Health, National University of Singapore, Singapore S117456, Singapore; 8 Institute for Digital Medicine, National University of Singapore, Singapore S117456, Singapore; 9 Singapore Institute for Clinical Sciences, Agency for Science, Technology and Research (A*STAR), Singapore 117609, Singapore

**Keywords:** SARS-CoV-2, temperature, universal health coverage, international tourism, elder

## Abstract

The current understanding of ambient temperature and its link to the outbreak of severe acute respiratory syndrome coronavirus 2 (SARS-CoV-2) is unclear. The objective of this study was to explore the environmental and climatic risk factors for SARS-CoV-2. For this study, we analyzed the data at the beginning of the outbreak (from 20 January to 31 March 2020) to avoid the influence of preventive or control measures. We obtained the number of cases and deaths due to SARS-CoV-2, international tourism, population age, universal health coverage, regional factors, the SARS-CoV-2 testing rate, and population density of a country. A total of 154 countries were included in this study. There were high incidence rates and mortality risks in the countries that had an average ambient temperature between 0 and 10 °C. The adjusted incidence rate for temperatures between 0 and 10 °C was 2.91 (95% CI 2.87–2.95). We randomly divided the data into a training set (80% of data) for model derivation and a test set (20% of data) for validation. Using a random forest statistical model, the model had high accuracy for predicting the high epidemic status of a country (ROC = 95.5%, 95% CI 87.9–100.0%) in the test set. Population age, temperature, and international tourism were the most important factors affecting the risk of SARS-CoV-2 in a country. An understanding the determinants of the SARS-CoV-2 outbreak can help to design better strategies for disease control. This study highlights the need to consider thermal effect in the prevention of emerging infectious diseases.

## 1. Introduction

Climate factors have been shown to influence the spread of infectious diseases. Since severe acute respiratory syndrome coronavirus 2 (SARS-CoV-2) initially emerged in China on December 31, correlations between climate factors and the spread of SARS-CoV-2 has been reported in many countries [1]. However, an association between temperature and the risk of SARS-CoV-2 remains unclear. In China, Qi et al. found that ambient temperature was negatively associated with the daily count of SARS-CoV-2 cases in the Hubei province of China (regression coefficient −3.6, *p*-value 0.01) [2]. A multicity study in China showed that each 1°C increase in ambient temperature was associated with a decline in daily confirmed case counts, with a corresponding overall relative risk of 0.80 (95% CI 0.75–0.85) [3]. In the USA, there were positive associations between the average temperature and total number of cases (Spearman’s correlation coefficient *r* = 0.379, *p*-value < 0.1) and mortality (*r* = 0.393, *p*-value < 0.1) in New York City [4]. In the Jakarta city in Indonesia, the average temperature significantly correlated with the SARS-CoV-2 pandemic (Spearman correlation coefficient *r* = 0.392, *p*-value < 0.01) [5]. To date, an association between temperature and the risk of the SARS-CoV-2 has been inconclusive.

The spread of SARS-CoV-2 could be affected by multiple factors [6,7]. In a population-based study, the level of migration was associated with the spread of SARS-CoV-2 in Hubei, China (Pearson’s correlation coefficient ranged from 0.61 to 0.84) [8]. Age and population density could influence the spread of the epidemic [9]. Universal health coverage (UHC) is an index of health coverage that includes financial risk protection, access to quality essential healthcare services, and access to safe, effective, high-quality, and affordable essential medicines and vaccines for all [10]. In a multinational comparison study, the UHC index was shown to be negatively associated with the incidence rate and mortality rate of infectious disease tuberculosis (*r* = −0.67 and *r* = −0.74, respectively) [11].

The objective of this study was to explore the association between the SARS-CoV-2 risk and ambient temperature and to correct for the effects of multiple factors to obtain global estimates. To avoid the impact of wearing masks, quarantine, lockdown, or vaccination, we analyzed global data from the early stages of the pandemic.

## 2. Materials and Methods

### 2.1. Study Countries and Number of Cases

We included all countries or territories that had reported confirmed SARS-CoV-2 cases from 20 January to 31 March 2020. The numbers of confirmed SARS-CoV-2 cases were obtained from the official counts in the WHO situation reports, in which the definition of a confirmed case was a person with laboratory confirmation of SARS-CoV-2 infection, irrespective of clinical signs and symptoms (https://www.who.int/, accessed on 31 March 2020). We excluded countries or territories that reported only cases under investigation or only had imported cases. We obtained the 2020 population data from the United Nations (UN) Department of Economic and Social Affairs [12]. We calculated the cumulative incidence and mortality due to SARS-CoV-2 between 20 January 2020 and 31 March 2020. This research was approved by the Research Ethics Committee of the National Taiwan University (no. 202004HM030).

### 2.2. Climate, Median Population Age, and International Tourism

We obtained climatic data from the World Meteorological Organization (http://data.un.org/, accessed on 31 March 2020) and the World Bank Group (https://climateknowledgeportal.worldbank.org/, accessed on 31 March 2020). We obtained the latest data and calculated the mean temperatures for January, February, and March between 1991 and 2016. The median population age (years) was obtained from the WHO, in which the population data were taken from the most recent UN Population Division’s “World Population Prospects” (https://www.un.org/development/desa/pd/, accessed on 31 March 2020). We obtained the latest data on the number of international tourist arrivals in 2018 from the World Tourism Organization (https://www.unwto.org/global-and-regional-tourism-performance/, accessed on 31 March 2020). The number of international inbound tourists (overnight visitors) was defined as the number of tourists who travelled to a country other than the country where they had their usual residence for a period not exceeding 12 months. We divided the number of international inbound tourists by the total population of the country, and obtained a proportion of the international tourist arrivals in each country.

### 2.3. Universal Health Coverage Index

The UHC index included the full spectrum of essential, quality health services, from health promotion to prevention, treatment, rehabilitation, and palliative care of a country [13]. We obtained the UHC index of the essential service coverage (%) of each country, from 2013 to 2017, from the WHO.

### 2.4. The SARS-CoV-2 Testing Rate

The number of confirmed cases might have been affected by the SARS-CoV-2 testing rate in each country. We obtained the total number of SARS-CoV-2 tests per 1000 people in each country from the online database of Our World in Data (https://ourworldindata.org/, accessed on 31 March 2020), which was hosted by researchers from the WHO, Johns Hopkins University, the University of Oxford, and other institutions. We obtained data between 21 January 2020 and 31 March 2020.

### 2.5. Population Density

We obtained the country population density from the Department of Economic and Social Affairs of the United Nations (https://population.un.org/wpp/, accessed on 31 March 2020). The summarized data can be downloaded from the 2020 World Population Review (https://worldpopulationreview.com/, accessed on 31 March 2020).

### 2.6. Statistical Analysis

Differences among groups were tested using the t-test and Mann–Whitney *U* test for means and medians, respectively, and the chi-square test for categorical variables. We used a scatterplot to show the temperature distribution and incidence of all countries. Then, we used Pearson’s correlation analysis to measure the direction of the relationship and the magnitude of the relationship between variables. We applied a multivariate Poisson regression model to adjust for confounders and calculated the incidence rate and mortality rate ratios. Because the association between temperature and risk had a nonlinear relationship, this finding could not have been inferred from the linear correlations alone. We applied random forest machine learning technology to model the relationship and assessed the accuracy of the model by the area under the receiver operating characteristic curves (AUC). We also applied the conditional inference trees to select important variables in the model [14]. A two-sided *p* < 0.05 was considered to be significant. Statistical analyses were done with R software (version 3.6.2) (R Foundation for Statistical Computing, Vienna, Austria) using functions from the packages of randomForest, ggplot2, ggpubr, ggrepel, corrgram, corrplot, pROC, and caret.

## 3. Results

**Figure 1:**
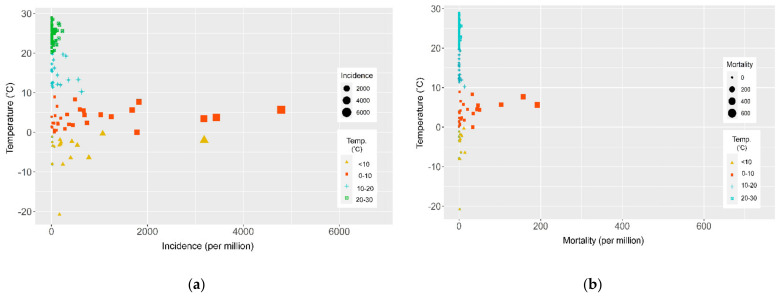
Scatterplot showing the relationship between average annual temperature (°C) and (**a**) incidence (per million) and (**b**) mortality (per million) of SARS-CoV-2.

A total of 202 countries/territories had reported 750,178 confirmed cases and 36,938 deaths of SARS-CoV-2 to the WHO by the end of March 2020. We excluded 48 countries that had only cases under investigation or imported cases; thus, a total of 154 countries/territories were included in this study. The global incidence rate of SARS-CoV-2 was 322.8 per million population. Europe had the highest incidence rate (784.8 per million), followed by the western Pacific region (102.1 per million), the region of the Americas (99.5 per million), the eastern Mediterranean region (84.6 per million), the African region (28.7 per million), and the South-East Asia region (9.3 per million). The global mortality rate of SARS-CoV-2 was 10.7 per million population. Europe had the highest mortality rate (28.8 per million). The scatterplots showed a unique distribution of high-risk countries with an average temperature between 0 and 10 °C (Figure 1). When we defined a high epidemic country as a country whose incidence rate was higher than the median incidence (60 per million), high epidemic countries had higher population age, population density, and international tourism arrivals than the low epidemic countries (Table 1).

**Figure 2:**
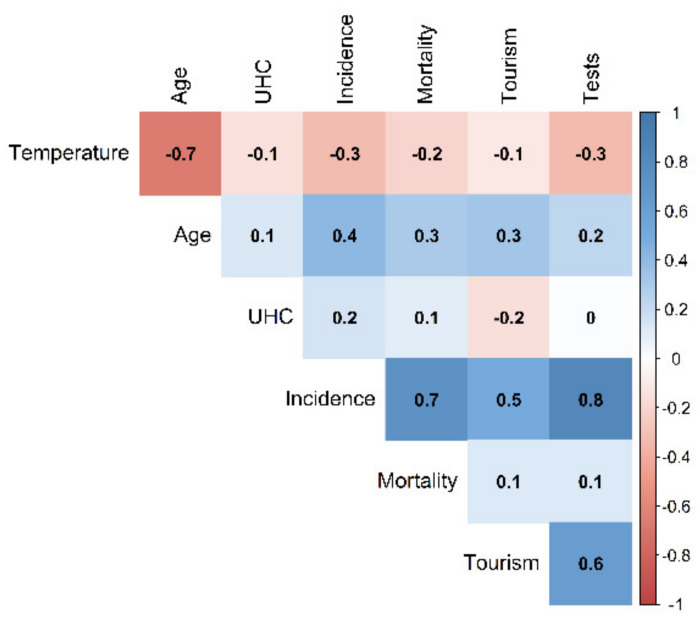
The Pearson correlation matrix of the ambient temperature (temperature), population age (age), universal health coverage (UHC), incidence rate (incidence), the mortality rate (mortality), the proportion of international tourism (tourism), and the SARS-CoV-2 testing rate (tests).

The correlation matrix showed a negative association between temperature and the incidence and mortality rates of SARS-CoV-2. The ambient temperature was negatively associated with the incidence rate (Pearson correlation coefficient, *r* = −0.33, *p*-value < 0.01) and mortality rate (*r* = −0.20, *p*-value = 0.02) of SARS-CoV-2. There was a high correlation between the SARS-CoV-2 testing rate and the incidence rate but not the mortality rate (Figure 2).

**Figure 3:**
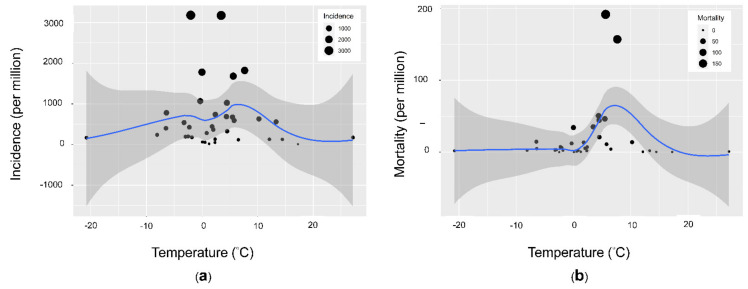
The nonlinear regression line of the average temperature and the (**a**) incidence rate and (**b**) mortality rate of SARS-CoV-2 in 36 OECD countries.

**Figure 4:**
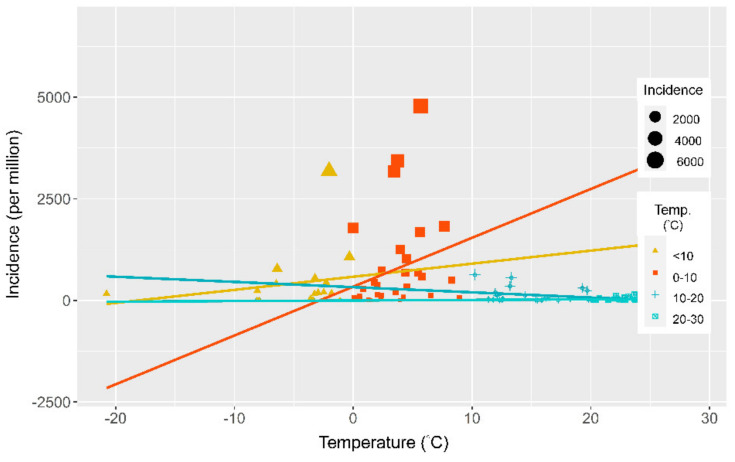
The incidence rates of four temperature groups.

**Figure 5:**
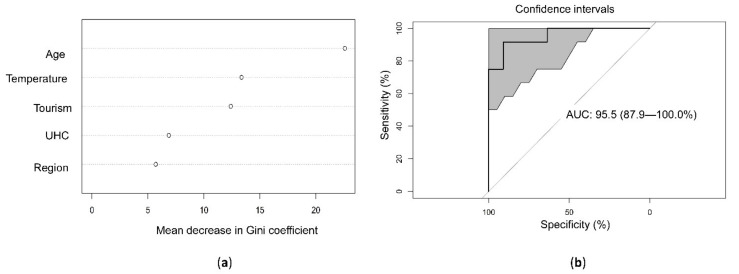
(**a**) Important variables for the incidence risk of severe acute respiratory syndrome coronavirus 2 modeled by random forest; (**b**) the accuracy of the prediction model, which is validated in the test set. The 95% confidence interval of receiver operating characteristic using bootstrap resampling for 2000 replicates was shown.

There were nonlinear relationships between ambient temperature and incidence and mortality. The incidence and mortality rates increased when the ambient temperature increased between 0 and 10 °C but decreased when the temperature was higher than 10 °C. The relationships were clear in the 36 Organization for Economic Cooperation and Development (OECD) countries that have similar economic and development levels. There was a nonlinear relationship between temperature and mortality. Mortality was positively associated with temperature when the temperature was between 0 and 10 °C but negatively associated with temperature when the temperature was higher than 10 °C (Figure 3). We included ambient temperature, relative humidity, health coverage, population age, population density, international tourism, regional factors, and the SARS-CoV-2 testing rate in the multivariate linear regression. Temperature and SARS-CoV-2 testing rate were the only significant factors that were positively associated with the incidence rate of SARS-CoV-2 (Table 2). We stratified the data by temperature to assess the effect of temperature on the risk of SARS-CoV-2. The countries with temperatures between 0 and 10 °C had the highest risk (Figure 4). Then, we divided the temperature into four groups (<0, 0–10, 10–20, and 20–30 °C) and used less than 0°C as the reference group to calculate the relative risk through the general linear model. The incidence rate ratio for ambient temperatures between 0 and 10°C was 2.91 (95% CI 2.87–2.95) (Table 3). We applied random forest statistics to build the prediction model for high epidemic countries. The independent variables included the country’s average temperature, relative humidity, population age, population density, universal health coverage, the proportion of international tourism arrivals, and the SARS-CoV-2 testing rate. We randomly divided the data into a training set (80% of data) for model derivation and a test set (20% of data) for validation. In the test set, the sensitivity was 0.9, specificity was 0.85, positive predictive value was 0.82, negative predictive value was 0.92, accuracy was 0.87 (95% CI 0.66–0.97), and kappa was 0.74. The area under the receiver operating characteristic curve in the test set showed a high accuracy of the model (ROC = 95.5%, 95% CI 87.9–100.0%). On the basis of the mean decrease in the Gini coefficient, the population age, temperature, and international tourism, were the most important factors that affected the epidemic status of a country (Figure 5).

## 4. Discussion

This study showed that there was a nonlinear association between the ambient temerature and the risk of SARS-CoV-2. After adjusting for the SARS-CoV-2 testing rate, population age, and the influences of international travel, the study showed an increased risk of SARS-CoV-2 at temperatures between 0 and 10 °C. This result is consistent with the findings of the United States and China at the beginning of the outbreak. In the United States, most cases were reported in states with a temperature between 4 and 11 °C [15]. In China, most cases were reported when the temperature was between 8 and 10 °C [16].

This study’s strength was that we applied the random forest statistical method to model the nonlinear association between temperature and the risk of the SARS-CoV-2 [14]. Infectious disease epidemics are known to be affected by environmental and social factors. The coefficient estimates of multiple linear regression may change erratically in response to small changes in the model or data owing to multicollinearity. Traditional regression models usually make assumptions about the distribution of data, but the associations between ambient temperature and the risk of SARS-CoV2 were nonlinear, and many independent variables were correlated. Problems have been observed in many studies that applied a traditional linear regression model to explore the association between temperature and SARS-CoV-2 (Supplementary Table S1). Machine learning statistical methods have advantages in creating sophisticated algorithms that can handle nonlinear data or problems of multicollinearity [17]. When we included the country’s average temperature, relative humidity, population age, population density, universal health coverage, the proportion of international tourism arrivals, and the SARS-CoV-2 testing rate in the machine learning model, the models had high accuracy in predicting the epidemic of SARS-CoV-2. Population age, temperature, and international tourism were important variables to predict the epidemic status. The finding is consistent with a population-based study in Iceland that reported the spread of SARS-COVID-2 was higher in higher age groups [18]. This study provided evidence that aging is a risk factor for SARS-CoV-2. We suggest that the government must give priority to vaccinating the elderly to reduce risks.

Coronavirus is a temperature-sensitive virus [19]. Under droplet transmission, respiratory viruses’ stability and viability are the highest in winter temperatures [20]. Coronavirus infection occurs more commonly in the winter and spring than in the summer and fall [21]. On the basis of experiences with the 2003 SARS outbreak, the transmission of coronavirus behaves seasonally and is dependent on seasonal temperature changes [22]. SARS-CoV-2 is a member of the same family of coronaviruses that caused severe acute respiratory syndrome (SARS) and Middle East respiratory syndrome (MERS) [23]. Droplet and airborne transmission are suspected to be the primary routes of SARS-CoV-2 virus transmission [24]. SARS-CoV-2 can be transmitted by indirect contact, and contaminated surfaces may play a significant role in infection transmission in the hospital and community. An in vitro study of SARS-CoV showed that virus viability was rapidly lost at higher temperatures and relative humidities [25]. Cai et al. showed that average temperature was inversely associated with SARS secondary attack rate [26]. In addition to transportation playing a central role in virus dispersal, low temperature may play a vital role in SARS-CoV-2 propagation. An experimental study showed that the infectivity of enveloped viruses in droplets or aerosols increased exponentially as the temperature decreased at a relative humidity of 75% [27], which is very close to our finding that the mean relative humidity in high epidemic countries was 76%.

Our analysis has limitations. First, an ecological study is an observational study that allows public health authorities to quickly adapt their strategies and efficiently organize a control response in the face of highly unpredictable events [28]. Ecological studies are commonly used in environmental health science since their unit of analysis is populations. However, ecological studies alone are insufficient for studying population health. The findings of an ecological study can only be used to generate hypotheses; no causal relationships should be inferred. Wearing a mask, isolation, lockdown, or vaccination can interrupt the spread of SARS-CoV-2. Therefore, this study analyzed the data at the beginning of the outbreak to prevent the influence of these preventive or control measures. High-endemic countries are defined as countries with an incidence rate higher than the median incidence rate (60 parts per million) of all countries in the early stages of the pandemic. The model obtained in this analysis should not be extended to risk prediction in other pandemic stages. 

## 5. Conclusions

Temperatures between 0 and 10 °C, higher population age, and international tourism are the most important risk factors for the disease outbreak. Understanding the determinants of the SARS-CoV-2 outbreak can help to design better strategies for disease control. This study highlights the need to consider climate factors in the prevention of emerging infectious diseases.

## Figures and Tables

**Table 1:** Characteristics by the epidemic status of the study countries.

Characteristics	High Epidemic ^⁋^ N = 77	Low Epidemic N = 77	*p*-Value ^¶^
Population age (years), mean (SD)	36.54 (5.36)	25.90 (7.33)	<0.01
Incidence (per million), median (IQR)	231.00 (411.00)	9.68 (15.90)	<0.01
Mortality (per million), median (IQR)	2.24 (5.86)	0.07 (0.40)	<0.01
Temperature (°C), median (IQR)	5.0 (16.5)	23.0 (10.3)	<0.01
Relative humidity (%), mean (SD)	76 (7)	68 (16)	0.03
Proportion of international tourism arrivals, median (IQR)	1.03 (1.81)	0.25 (0.39)	<0.01
Universal health coverage, mean (SD)	0.44 (0.07)	0.43 (0.08)	0.44
SARS-CoV-2 testing rate per 1000 people, median (IQR)	28.20 (64.90)	1.23 (3.58)	<0.01
Population density per km^2^, median (IQR)	111.00 (250.00)	72.00 (107.00)	<0.01
Region (no.)			<0.01
Africa	3	22	
Eastern Mediterranean	6	11	
Europe	43	11	
Americas	17	18	
South-East Asia	0	7	
Western Pacific	8	7	

^⁋^ A high epidemic country was defined as one in which the incidence rate was higher than the median incidence rate (60 per million) of all countries. ^¶^ Statistical tests performed included the t-test and Mann–Whitney *U* test for means and medians, respectively, and the chi-square test for categorical variables. IQR, interquartile range; SD, standard deviation; no., number.

**Table 2:** The parameter estimates of the multiple linear regression models.

Variable	Incidence ^⁋^		
	Estimate	S.E.	*p*-Value
Intercept	−582.74	392.33	0.14
Average temperature	14.5	5.2	0.01
Universal health coverage	−275.90	600.37	0.65
Population median age	17.46	8.69	0.05
Proportion of international tourism arrivals	−36.99	37.34	0.33
Region			
Eastern Mediterranean	−94.20	233.07	0.69
Europe	293.95	229.30	0.21
Americas	−34.86	190.38	0.86
South-East Asia	−160.82	235.54	0.50
Western Pacific	−231.14	226.56	0.31
SARS-CoV-2 testing rate	5.37	0.57	<0.01

**^⁋^** Multiple R^2^ was 0.80, and adjusted R^2^ was 0.76.

**Table 3:** The incidence rate ratio of SARS-CoV-2 according to temperature.

Variable	RR	LCI	UCI
Average temperature			
0–10 °C	2.91.	2.87	2.95
10–20 °C	0.36	0.35	0.37
20–30 °C	0.10	0.10	0.10
SARS-CoV-2 testing rate	1.01	1.00	1.01
Population age	1.11	1.11	1.11
Proportion of international tourism arrivals	1.31	1.31	1.32

Abbreviations: RR, relative risk; UCI, upper confidence interval; LCI, lower confidence interval. The null deviance of the model was 1,257,471 on 55 degrees of freedom, residual deviance was 286,077 on 49 degrees of freedom, and the Akaike information criterion (AIC) was 286,597.

## Data Availability

The open data used in this study are available online.

## Supplementary Materials

The following are available online at https://www.mdpi.com/article/10.3390/ijerph18084052/s1, Table S1: Systemic review of the association between ambient temperature and SARS-CoV-2 using the Newcastle Ottawa Scale.
