# Supplementary material for: The Impact of Temperature on the Risk of COVID-19: A Multinational Study
Source: Int J Environ Res Public Health. 2021 Apr 12;18(8):4052. doi: 10.3390/ijerph18084052 (PMC8068915; doi:10.3390/ijerph18084052)
Supplement: Supplementary file 1 [file ijerph-18-04052-s001.pdf]

Table S1. Systemic Review of the Association between Ambient Temperature and SARS-CoV-2 Using the Newcastle Ottawa Scale

	Country/area	Climatic data and source	Main findings	Advantages	Limitations	Newcastle Ottawa Score
Ahmadi et al. [1]	Iran	Average temperature, average precipitation, humidity, wind speed, average solar radiation: Weather Spark online web service: https://weatherspark.com/	Population density was the most correlated variable (regression coefficient $r = 0.523$, P-value = 0.00), followed by movement ($r = 0.232$, P-value = 0.003). Average temperature had positive correlation with infection rate ($r = 0.013$, P-value = 0.872). The humidity ($r = -0.160$, P-value = 0.08) and wind speed ($r = -0.089$, P-value = 0.34) had negative correlation with infection rate.	Consider population density and intra-provincial movement	The source of the intra-provincial movement, the method to calculate the average climatic data was unclear.	5
Bashir et al. [2]	New York City, US	National weather service, USA	Temperature minimum had positive correlation with the number of daily new cases ($r = 0.248$, P-value < 0.05); average temperature was associated with mortality ($r = 0.294$, P-value < 0.05). Air quality was negatively correlated with daily new cases and mortality.	Considered the air quality	NA	5
Briz-Redon A et al. [3]	Spain	Daily mean, minimum and maximum temperatures: OpenData platform of the State Meteorological Agency (AEMET) (http://www.aemet.es/en/portada)	No relationship between the number of SARS-CoV-2 cases and temperature.	Consider population density, age, number of travelers and number of companies; use of spatio-temporal model to adjust the lag effect	NA	7
Del Rio C et al. [4]	China, Italy, South Korea, Iran, Philippine, Belgium,	Daily average, maximum, and minimal ambient temperatures: Weather Channel website (https://weather.com/)	The regions with ongoing human-to-human transmission showed significantly higher temperatures (5.14 ± 5.94℃) than the regions without ongoing human-to-human	The only study that compared temperature in regions with/without a	The way to determine region with human to human transmission was unclear	4

	Finland, Egypt, Australia		transmission (17.06 ± 11.94℃) (*P*-value < 0.05).	human to human transmission.		
Demongeot J at al. [5]	21 countries	1. Weather Atlas (https://www.weather-atlas.com) or UNdata (http://data.un.org/Data.aspx?d=CLINO&f=ElementCode%3A11).<br>2. Mean annual temperatures for that country: Wikipedia (https://en.wikipedia.org/wiki/List_of_countries_ by_average_yearly_temperature).	High temperatures diminish initial contagion rates, but seasonal temperature effects at later stages of the epidemy remain questionable.	Only study that used the ARIMA regression to approach the new cases' time series in five countries with different weather.	Consider the temperature only	4
Gunthe SS et al. [6]	Global	Minimum, maximum, and average temperature; relative humidity, UV index, precipitation, and cloud cover: World Weather Online (https://www.worldweatheronline.com)	There is a very narrow temperature band of 3–12 °C, where 90% of the total confirmed cases are agglomerated.	NA	The way to calculate the mean temperature of a country is unclear.	5
Gupta S et al. [7]	US	Daily temperature, relative humidity: the National Centers for Environmental Information (NCEI) library (https://www.ncdc.noaa.gov/data-access)	The majority of the cases were reported in states experiencing temperatures of 4–11 °C and absolutete humidity of 4–6 g/m3.	Indicated absolute humidity to be a better metric than relative humidity and temperature to study the spread.	NA	6
Jahangiri M et al. [8]	Iran	Iran Meteorological Organization (IMO) (http://www.irimo.ir/far/index.php).	AUC of the ambient temperature model was 0.5.	NA	The way to build the model and the dependent variablwasre unclear.	4
Pirouz B, et al.[9]	China, Japan, South Korea, Italy	Daily wind, humidity, average temperature: unclear source	Relative humidity with an average of 77.9% affected positively, and maximum daily temperature with an average of 15.4 °C affected negatively on the confirmed cases. High predictive accuracy of the model.	Provided predictive accuracy using a testing set.	It is difficult to independently verify the quality and accuracy of the data.	4
Pirouz B, et al.[10]	Italy	Average temperature, humidity, wind: unclear source	Weather conditions could affect the daily positive cases	NA	It is difficult to independently verify the quality	4

					and accuracy of the data.	
Prata DN, et al. [11]	Brazil	Annual average temperature: National Institute of Meteorology authority in Brazil	Whe the temperature was below 25.8°C, each 1°C rise was associated with a −4.8951% decrease in the number of daily cumulative confirmed cases	NA	It is difficult to independently verify the quality and accuracy of the data.	3
Sahin M., et al.[12]	Turkey	Temperature, dew point, humidity, and wind speed: Weather Underground (https://www.wunderground.com/)	Temperature on the day of cases has the highest correlation with the cases (r = -0.483).	NA	The way to calculate the mean temperature is unclear.	3
Shi, P. et al.[13]	China	Daily mean temperature: meteorological authority in mainland China (http://data.cma.cn).	There was a biphasic relationship of daily confirmed cases rate with temperature (with a peak at about 10 °C).<br>Higher temperatures were associated with a lower relative risk (RR) was 0.96 (95% CI 0.93–0.99).	Consider exposure-lag-response association; conservative explain the results and limitations of the study.	NA	8
Sobral MFF, et al.[14]	Global	Precipitation, average temperature, maximum temperature, and minimum temperature: the National Oceanic and Atmospheric Administration (NOAA) database	Negative association between temperature and the numberer of cases.	Demographic density of each country was used as a control variable, with data obtained from the Food and Agriculture Organization and World Bank population estimates for the year 2018 (World Bank 2020).	Collinearity: put highly correlated variables of average, maximum, and minimum temperatures into the regression model	7
Tobias A, et al.[15]	Spain	Daily maximum temperature: the Meteorological Service of Catalunya (MeteoCat) (https://en.meteocat.gencat.cat/?lang=en)	An average increase of 1ºC of maximum temperature decreased the incidence rate by -7.5% (95% CI -12.3– -2.6).	Adjusted for time-trends and accounted for residual autocorrelation	Small number of days	4

Tosepu R, et al.[16]	Indonesia	Temperature minimum, temperature maximum, temperature average, humidity, rainfall: Meteorological Department of the Republic of Indonesia	Temperature average was correlated with the number of cases (r = 0.392, P-value < 0·01).	NA	NA	4
Ujiie M, et al.[17]	Japan	Japan Meteorological Agency: (https://www.data.jma.go.jp/obd/stats/etrn/index.php)	Regression coefficient of temperature was -0.128 (P-value < 0.01).	Adjusted for inbound visitors from China and the ratio of elder people aged ≥ 65	NA	
Yao Y, et al.[18]	China	1. Daily mean temperature, relative humidity: China Meteorological Data Sharing Service System.<br>2. UV radiation: Dutch–Finnish ozone monitoring instrument (OMI) level 2 UV irradiance products, version 003 (OMUVB V003) (https://disc.gsfc.nasa.gov/datasets/OMUVB_003/summary)	After adjustment for relative humidity and UV, there was no association of transmission with temperature or UV radiation in Chinese cities.	The only study that analyzed the association between basic reproduction number (R0) and temperature.	R0 is a dynamic number that will change over time. The study did not explain how did they calculate the R0 in different dates and cities.	5
Liu J, et al.[19]	China	Hourly temperature, relative humidity: Shanghai Meteorological Bureau and Data Center of Ministry of Ecology (absolute humidity was calculated by vapor pressure)	Each 1 °C increase in ambient temperature was related to the decline of daily confirmed case counts, and the corresponding pooled RRs were 0.80 (95% CI 0.75–0.85).	Considered the population migration and lag effect. Used meta-analysis to pool effect in 30 cites.	Did not consider the number of population in each city	7
Qi H, et al.[20]	China	Daily average ambient temperature, relative humidity: Weather Underground (https://www.wunderground.com/)	Every 1 °C increase in ambient temperature led to a decrease in the daily confirmed cases by 36% to 57% when relative humidity was in 67% to 85.5%.	Showed interaction between average temperature and relative humidity on SARS-CoV-2.	Use of the Baidu index as a measure of health-seeking behavior needs to be validated.	6
Wu Y, et al.[21]	Global	1. Daily average temperature, average dew point, and average wind speed: National Oceanic and Atmospheric Administration Center (https://www.ncei.noaa.gov/access/s	A 1 °C increase in temperature was associated with a 3.08% (95% CI 1.53–4.63%) reduction in daily new cases and a 1·19% (95% CI 0.44– 1.95%) reduction in daily new deaths.	Multi-national comparison	The reason to exclude China in the analysis is unclear.	7

earch/data-search/global-summary-of-the-day).
2. Relative humidity: calculated from the vapor pressure of air at a temperature and saturation water vapor pressure, source unclear.
3. Wind speed: National Oceanic and Atmospheric Administration Center (https://www.ncei.noaa.gov/access/search/data-search/global-summary-of-the-day)

1. Ahmadi, M.; Sharifi, A.; Dorosti, S.; Jafarzadeh Ghoushchi, S.; Ghanbari, N. Investigation of effective climatology parameters on COVID-19 outbreak in Iran. The Science of the total environment 2020, 729, 138705, doi:10.1016/j.scitotenv.2020.138705.
2. Bashir, M.F.; Ma, B.; Bilal; Komal, B.; Bashir, M.A.; Tan, D.; Bashir, M. Correlation between climate indicators and COVID-19 pandemic in New York, USA. Sci Total Environ 2020, 728, 138835, doi:10.1016/j.scitotenv.2020.138835.
3. Briz-Redon, A.; Serrano-Aroca, A. A spatio-temporal analysis for exploring the effect of temperature on COVID-19 early evolution in Spain. The Science of the total environment 2020, 728, 138811, doi:10.1016/j.scitotenv.2020.138811.
4. Del Rio, C.; Camacho-Ortiz, A. Will environmental changes in temperature affect the course of COVID-19? The Brazilian journal of infectious diseases : an official publication of the Brazilian Society of Infectious Diseases 2020, 10.1016/j.bjid.2020.04.007, doi:10.1016/j.bjid.2020.04.007.
5. Demongeot, J.; Flet-Berliac, Y.; Seligmann, H. Temperature decreases spread parameters of the new Covid-19 case dynamics. Biology 2020, 9, doi:10.3390/biology9050094.
6. Gunthe, S.S.; Swain, B.; Patra, S.S.; Amte, A. On the global trends and spread of the COVID-19 outbreak: preliminary assessment of the potential relation between location-specific temperature and UV index. Journal of Public Health-Heidelberg 2020, Published online April 24, doi:10.1007/s10389-020-01279-y.
7. Gupta, S.; Raghuwanshi, G.S.; Chanda, A. Effect of weather on COVID-19 spread in the US: A prediction model for India in 2020. Sci Total Environ 2020, 728, 138860, doi:10.1016/j.scitotenv.2020.138860.
8. Jahangiri, M.; Jahangiri, M.; Najafgholipour, M. The sensitivity and specificity analyses of ambient temperature and population size on the transmission rate of the novel coronavirus (COVID-19) in different provinces of Iran. Sci Total Environ 2020, 728, 138872, doi:10.1016/j.scitotenv.2020.138872.
9. Pirouz, B.; Haghshenas, S.S.; Haghshenas, S.S.; Piro, P. Investigating a serious challenge in the sustainable development process: analysis of confirmed cases of COVID-19 (new type of coronavirus) through a binary classification using artificial intelligence and regression analysis. Sustainability 2020, 12, doi:10.3390/su12062427.
10. Pirouz, B.; Shaffiee Haghshenas, S.; Pirouz, B.; Shaffiee Haghshenas, S.; Piro, P. Development of an assessment method for investigating the impact of climate and urban parameters in confirmed cases of COVID-19: a new challenge in sustainable development. International journal of environmental research and public health 2020, 17, doi:10.3390/ijerph17082801.
11. Prata, D.N.; Rodrigues, W.; Bermejo, P.H. Temperature significantly changes COVID-19 transmission in (sub)tropical cities of Brazil. The Science of the total environment 2020, 729, 138862, doi:10.1016/j.scitotenv.2020.138862.
12. Sahin, M. Impact of weather on COVID-19 pandemic in Turkey. Sci Total Environ 2020, 728, 138810, doi:10.1016/j.scitotenv.2020.138810.
13. Shi, P.; Dong, Y.; Yan, H.; Zhao, C.; Li, X.; Liu, W.; He, M.; Tang, S.; Xi, S. Impact of temperature on the dynamics of the COVID-19 outbreak in China. Sci Total Environ 2020, 728, 138890, doi:10.1016/j.scitotenv.2020.138890.
14. Sobral, M.F.F.; Duarte, G.B.; da Penha Sobral, A.I.G.; Marinho, M.L.M.; de Souza Melo, A. Association between climate variables and global transmission oF SARS-CoV-2. The Science of the total environment 2020, 729, 138997, doi:10.1016/j.scitotenv.2020.138997.
15. Tobias, A.; Molina, T. Is temperature reducing the transmission of COVID-19 ? Environ Res 2020, 186, 109553, doi:10.1016/j.envres.2020.109553.
16. Tosepu, R.; Gunawan, J.; Effendy, D.S.; Ahmad, O.A.I.; Lestari, H.; Bahar, H.; Asfian, P. Correlation between weather and Covid-19 pandemic in Jakarta, Indonesia. Sci Total Environ 2020, 725, 138436, doi:10.1016/j.scitotenv.2020.138436.
17. Ujiie, M.; Tsuzuki, S.; Ohmagari, N. Effect of temperature on the infectivity of COVID-19. International journal of infectious diseases : IJID : official publication of the International Society for Infectious Diseases 2020, 10.1016/j.ijid.2020.04.068, doi:10.1016/j.ijid.2020.04.068.

18. Yao, Y.; Pan, J.; Liu, Z.; Meng, X.; Wang, W.; Kan, H.; Wang, W. No association of COVID-19 transmission with temperature or UV radiation in Chinese cities. Eur Respir J 2020, 55, doi:10.1183/13993003.00517-2020.
19. Liu, J.; Zhou, J.; Yao, J.; Zhang, X.; Li, L.; Xu, X.; He, X.; Wang, B.; Fu, S.; Niu, T., et al. Impact of meteorological factors on the COVID-19 transmission: A multi-city study in China. Sci Total Environ 2020, 726, 138513, doi:10.1016/j.scitotenv.2020.138513.
20. Qi, H.; Xiao, S.; Shi, R.; Ward, M.P.; Chen, Y.; Tu, W.; Su, Q.; Wang, W.; Wang, X.; Zhang, Z. COVID-19 transmission in Mainland China is associated with temperature and humidity: A time-series analysis. Sci Total Environ 2020, 728, 138778, doi:10.1016/j.scitotenv.2020.138778.
21. Wu, Y.; Jing, W.; Liu, J.; Ma, Q.; Yuan, J.; Wang, Y.; Du, M.; Liu, M. Effects of temperature and humidity on the daily new cases and new deaths of COVID-19 in 166 countries. The Science of the total environment 2020, 729, 139051, doi:10.1016/j.scitotenv.2020.139051.
